# Composite hemangioendothelioma of the forehead and right eye; a case report

**DOI:** 10.1186/s12895-017-0067-4

**Published:** 2017-12-12

**Authors:** Ghasem Rahmatpour Rokni, Fatemeh Montazer, Mahnaz Sharifian, Mohamad Goldust

**Affiliations:** 1 Dermatology, Clinical Research Development Unit of Bou-Ali Sina Hospital, Mazandaran University of Medical Sciences, Sari, Iran; 2 Department of Pathology, Gastrointestinal Cancer Research Center, Mazandaran University of Medical Sciences, Sari, Iran; 3 Department of Pathology, Babol University of Medical Sciences, Babol, Iran; 4 Tabriz University of Medical Sciences, Tabriz, Iran

**Keywords:** Hemangioendothelioma, Vascular neoplasm, Composite hemangioendothelioma

## Abstract

**Background:**

Hemangioendothelioma includes several types of vascular neoplasms, as well as both benign and malignant growth.

**Case presentation:**

This study evaluated a case of a 78-year-old female diagnosed with composite hemangioendothelioma (CHE). This patient had an 18-month history of painless inflammatory lesions and erythema on the left forehead and right upper eyelid. The clinical and pathologic characteristics of the CHE were evaluated in the present study.

**Conclusion:**

The evidence of the coexistence of variable components of the CHE in our study emphasized the importance of distinctive morphology and biology, and should be considered in the differential diagnosis of other vascular lesions.

## Background

Composite hemangioendothelioma (CHE) is a type of recently described vascular tumor named hemangioendothelioma (HE). This may be benign or malignant depending on the particular group member’s function. This lesion is known as a low-grade malignant neoplasm. In most cases, the CHE are solitary lesions that occur as dermal papules or nodules; however, they may be multiple. The colors of these lesions are commonly red, purple, or black violaceous, and they can be scaly or hemorrhagic [1, 2]. According to previous studies, there is an association between the CHE and some tumors (such as vascular tumors) and hemangioma [3]*.* The hemangioendothelioma was initially considered a type of vascular neoplasm with different combinations and scopes, varying from hemangiomas to highly malignant angiosarcomas. Owing to the morphologic heterogeneity of CHE, however, it was introduced as a new diagnosis. This tumor has different types of grading, including benign, low-grade malignant, and malignant. The differential diagnosis of CHE is crucial due to the morphologic heterogeneity of CHE and it plays an important role in the careful evaluation of CHE. A biopsy test result is very important to determine the combination of vascular components and the features of individual vascular patterns [4]. This study described a case of CHE arising on the face and scalp area of a 78-year-old female. The clinical and pathological characteristics of this CHE were evaluated. This study emphasized the importance of an accurate diagnosis of CHE in order to ensure correct management.

## Case presentation

**Fig. 1:**
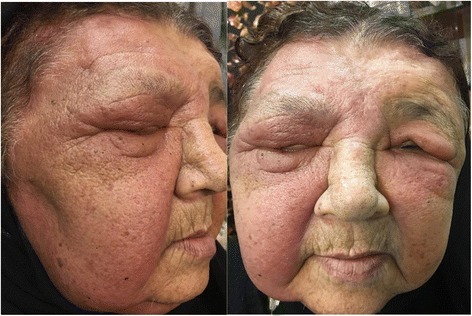
The swelling of the eyelids resulted in complete closure of the eyelids on both sides

**Fig. 2:**
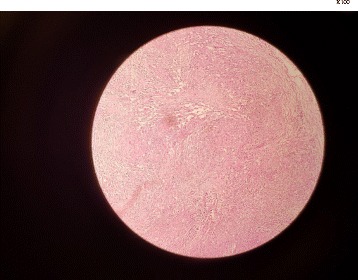
Both component o epithelioid hemangioenthelial and angiosarcoma

**Fig. 3:**
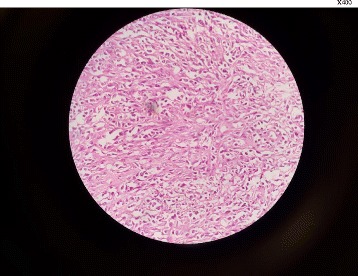
Angiosarcoma component

**Fig. 4:**
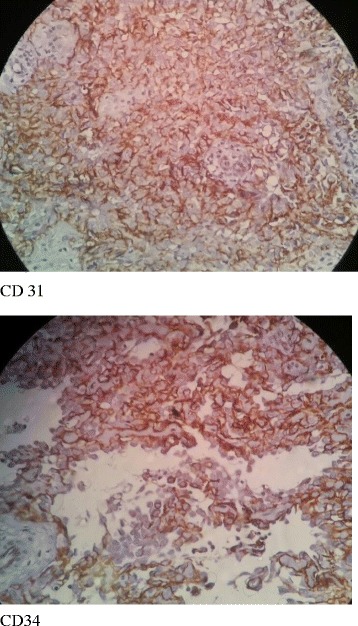
Diffuse and dense staining for cd31 cd34 in epitheliod and vascular component

This study presented a case of a 78-year-old female with an 18-month history of painless progressive swelling and erythema on the left forehead and right upper eyelid. The lesions were slightly developed and involved all of the scalp surface, eyelids, and face; however, there was no other abnormality. The chief complaint of the patient was the mass on the right eyelid. She had a proptosis of the right eye and an orbital mass; however, there were no signs of headache, nausea, vomiting, or double vision. The medical history of the patient demonstrated that she had hypertension and coronary artery disease, and had undergone angioplasty. However, she had no history of undergoing radiotherapy or chemotherapy. The result of the brain MRI in May 2015 demonstrated a mucosal thickening in the right maxillary sinus. A soft tissue with a slightly lobulated surface was observed using magnetic resonance imaging (MRI). The ventricles, subarachnoid space, and brain stem demonstrated normal appearance, and there was no evidence of mass lesion or midline shift. The MRI with and without contrast, performed 2 months later, revealed a tissue measuring 5 × 3 × 1 cm and weighing 12 g in the right orbital mass. The microscopic findings demonstrated a benign lesion composed of a mixture of angiomatous tissue and lobulated fatty tissue connected with skeletal muscle tissue, which was suggestive of hemangioma. The brain computed tomography (CT) findings showed that there were no abnormalities in the brain structure. There was diffused skin and subcutaneous soft tissue edema in the cranial area, which was more pronounced in the radiation therapy site. The ultrasound reviewed an inflammatory soft tissue in the right frontotemporal region progressing to the ear. The patient used atenolol 50 mg (twice daily), triamterene-h (half a pill every 12 h), acetylsalicylic acid (daily), and atorvastatin 20 mg (daily). In July 2016, she was hospitalized in the infectious diseases ward with a diagnosis of cellulitis and received oral antibiotic therapy (cephalexin) for 2 weeks. Owing to the lack of response to the treatment, she was then referred to the dermatology department. Swelling and slightly warm soft tissue along with erythema was observed throughout the facial surface and scalp. The swelling of the eyelids resulted in complete closure of the eyelids on both sides, which impaired the patient’s vision; however, the patient’s eye exam was normal (Fig. 1). The complete blood count test was done for the patient (hemoglobin level: 14.7 g/dl, white blood cells: 5950 cells/mm3, neutrophils: 50%, lymphocytes: 35%, monocytes: 10%, eosinophils: 1%, basophils: 3%, sodium: 141 mEq/L, potassium: 3.8 mmol/L, blood urea nitrogen: 53 mg/dL, creatinine: 1.2 mg/dL, blood sugar: 163 mg/dL). Furthermore, the urinalysis and liver enzyme test demonstrated normal results. Based on the CT scan with and without contrast, no anomalies or bone lesions were reported. The brain MRI showed that there was an area of increased signal intensity on the axial T2-weighted images in the skin and subcutaneous tissue of the right cheek. Five months later, an elliptical biopsy at the margin of the lesion was done and the microscopic examination revealed a malignant vascular neoplasm composed of a poorly circumscribed dermal and subcutaneous tumor with an infiltrative growth pattern. In some areas, there were proliferations of solid epithelioid cells with vesicular nuclei, eosinophilic cytoplasm, and intracytoplasmic lumina, which intermixed through vascular channels with retiform architecture lined by plump hyperchromatic endothelial cells with a hobnail appearance. The lining of endothelial cells in some areas showed multi-layering plump and occasionally pleomorphic nuclei with scattered mitotic figures (Figs 2 and 3). The diagnosis was a low-grade malignant vascular neoplasm compatible with CHE. The patient was referred to undergo the immunohistochemistry (IHC) test. The IHC test demonstrated negative results regarding the tumor cell in MNF 116 and SMA; however, it showed positive results for CD31 and CD34 (Fig. 4). Furthermore, Ki67 was observed to be positive in only 8% of the tumor cells. Based on the combination of histopathological findings and IHC results, the patient was diagnosed with CHE; therefore, she was recommended to undergo a complete lesion excision due to the involved cervical lymph node. The patient underwent partial excision of lesions and skin grafting. Post-surgery, she received chemotherapy by 100 mg thalidomide (daily). Following the thalidomide treatment, the erythema and swelling regressed, and she was able to open the eyelids.

## Discussion

Composite Hemangioendothelioma (CHE) is a new entity classified under the Heman gioendothelioma (HE) group of tumours. It is an extremely rare vascular neoplasm of low to moderate malignancy. Based on the previous studies, the CHE may present at any age and its size ranges from 0.7–30 cm. These lesions are usually composed of circumscribed nodules, plaques, or ulcerated tumors. The risk of CHE was reported to be higher in females than males [5, 6]. It commonly occurred on the dermis or subcutaneous tissues in over half of the reported cases. However, the CHE may occur in different locations of the body. The head and neck lesions are the second-most-common sites of CHE development. In our case, the CHE was limited to the facial and scalp surface. The lesion of our study was unique since it involved all of the scalp surface, eyelids, and face and the swelling of the eyelids resulted in complete closure of the eyelids on both sides, which impaired the patient’s vision; however, the patient’s eye examination was normal. It seems that the CHE is a lesion composed of a mixture of different patterns, which can vary from tumor to tumor [7, 8]. In the CHE, each component has different characteristics, and the ratio and distribution of each component are diverse in each lesion. As observed in our study, retiform HE is the most common histological component in the CHE in which the branching blood vessels with slender and anastomosing walls are observable and resemble the rete testis. In most patients with CHE, the predominant components are composed of epithelioid HE and spindle cell hemangioma. Nevertheless, papillary structures such as papillary intralymphatic angioendothelioma may be observed in some cases. Although the CHE has regions of benign component, it can have the characteristics of an arteriovenous malformation and cavernous hemangioma, as indicated in some studies [9, 10]. The angiosarcoma-like regions are also observed in approximately half of the CHE cases; however, high-grade angiosarcoma, in which the solid sums of abnormal pleomorphic cells and numerous mitotic shapes are observed, is rare. The diagnosis of CHE in the head and neck should be distinguished from a number of vascular tumors that occur in these regions. Although the CHE may contain angiosarcoma-like regions, it should be differentiated from pure angiosarcomas [11, 12]. Angiosarcomas of the head and neck (particularly on the scalp and face) usually occur in elderly patients. Since our case was an elderly woman, the possibility of misdiagnosis was high. The epithelioid angiosarcoma shows diffuse rather than localized nuclear atypia; furthermore, it includes more marked mitotic function than the CHE. Angiosarcoma-like foci and a prior history of lymphedema are presented in the majority of CHE patients, which can be indicative of low-grade angiosarcoma. The prognosis of CHE is partly better than that of the conventional angiosarcoma; therefore, it must be classified as a distinct vascular tumor. Moreover, the epithelioid HE occurs in the head and neck areas; however, it may present multi-centrically, and usually occurs in the deep soft tissues, solid organs, and bones. The CHE is mainly located at the cutaneous level; nevertheless, the presentation of epithelioid HE on the skin is rare [13]. The differential diagnosis of CHE is very important due to the morphologic heterogeneity of this lesion. Based on the confined or non-representative sampling, even the diagnosis based on core biopsy can be indefinite. Therefore, a careful evaluation of the biopsy test results is very important to determine the combination of vascular components and merging features of the vascular patterns. A biopsy can be helpful to distinguish the CHE from other vascular lesions. IHC studies have shown that some endothelial cell markers are partly negative or weakly positive in the cell proliferation of CHE [14]. In our study, the IHC test revealed negative results regarding the tumor cell in MNF 116 and SMA, and Ki67 was observed to be positive in only 8% of the tumor cells. The CHE may be mistaken for polymorphous HE; therefore, its diagnosis should be applied with caution. CHE lesions have various histopathological patterns, whereas the polymorphous HE is specific to the soft tissues and lymph nodes. Histopathologically, there are a combination of undifferentiated solid regions in the polymorphous lesions, in which an angiomatous pattern and uniform cytological elements are observed [15]. The treatments of the CHE and epithelioid HE of soft tissue are similar. The surgical excision is the routine treatment of the CHE; however, there are patients who had been treated with interferon and electron beams. Although these methods are used in the treatment of the CHE, the patients undergoing surgery are reported to have better prognosis and lower recurrence rates. The CHE recurrence is observed in about half of the cases; nonetheless, no deaths have occurred as a result of these lesions. Owing to the recurrence propensity of the CHE and its potential to metastasize, the prevention of the relapse of these lesions is critical [16]. Thalidomide was first used as an antiemetic. Given the mechanism of action against angiogenesis, thalidomide has a valid role in vascular tumors. In their study Salech et al. evaluated the thalidomide for the treatment of metastatic hepatic epithelioid hemangioendothelioma and demonstrated that the drug was well tolerated with minimal toxicity and the patient continues on therapy 109 months after treatment was started with no disease progression [17]. Soape et al. evaluated the efficacy of thalidomide in the treatment of hepatic epithelioid hemangioendothelioma and stated that the use of thalidomide as chemotherapy is novel and promising, especially in the setting of a rare vascular tumor such as hemangioendothelioma [18]. In contrary to previous studies, our study demonstrated that since thalidomide led to reducing in periorbital edema and eliminating the lesion pressure effect the tumor did not regress completely.

## Conclusion

In our study, there were various components of the CHE and it was unique due to site and extent of the lesion. Therefore, the diagnosis of the CHE features is very important and should be considered in the differential diagnosis of other vascular lesions.

## Abbreviations

CHE: Composite hemangioendothelioma
CT: Computed tomography
HE: Hemangioendothelioma
IHC: Immunohistochemistry
MRI: Magnetic resonance imaging
